# VZV Prophylaxis After Allogeneic Hematopoietic Stem Cell Transplantation in Children: When to Stop?

**DOI:** 10.1002/cnr2.70015

**Published:** 2024-11-07

**Authors:** Eva de Berranger, Anne‐Flore Derache, Nassima Ramdane, Julien Labreuche, Pauline Navarin, Fanny Gonzales, Wadih Abou‐Chahla, Brigitte Nelken, Bénédicte Bruno

**Affiliations:** ^1^ Paediatric Hematology Lille France; ^2^ Department of Biostatistics Lille France

**Keywords:** acyclovir prophylaxis, allogeneic hematopoietic stem cell transplantation, children, herpes zoster, lymphocytes immunophenotyping, VZV infection

## Abstract

**Background:**

Acyclovir treatment is an efficient prophylaxis to prevent varicella‐zoster virus (VZV) reactivation after allogeneic hematopoietic stem cell transplantation (HSCT).

**Aims:**

This single center retrospective study tried to determine if the lymphocytes immunophenotyping could help to determine the duration of prophylaxis, and evaluated complications, and associated risk factors for VZV infection.

**Methods and Results:**

Eighty‐four children underwent an allogeneic HSCT, in which 77 received an acyclovir prophylaxis. Twenty‐one of the 77 had a VZV infection with an incidence rate of 1.30 per 100 patients‐months (exact 95% CI, 0.81 to 2.01). Among these 21 patients with VZV infection, 16 had an infection after withdrawing acyclovir prophylaxis within a median of 49 days (range, 11 days–5.8 months). Thirty‐five percent of the VZV infected patients were hospitalized, 9% had a visceral dissemination, and 9% had postherpetic neuralgia. In multivariate analysis, higher VZV infection rate was associated with conditioning regimen with total body irradiation, immunoglobulin substitution, and antithymocyte globulin. The incidence of VZV infection increased significantly when patients had a CD4+ lymphocytes count below 23% (cHR 3.28 [95% CI, 1.09–9.81]; *p* = 0.03) or a CD4^+^/CD8^+^ ratio less than 0.9 (cHR 3.13 [95% CI, 1.04–9.36]; *p* = 0.04) at the time of stopping acyclovir prophylaxis.

**Conclusion:**

After cessation of acyclovir prophylaxis, VZV reactivation can occur and be responsible for morbidity after allogeneic HSCT. This study suggests that the proportion of CD4+ lymphocytes and the CD4^+^/CD8^+^ ratio can inform decisions about the duration of acyclovir prophylaxis after allogeneic HSCT to prevent VZV reactivation.

## 1 Introduction

Varicella‐zoster virus (VZV) reactivation is a significant cause of morbidity and mortality after allogeneic hematopoietic stem cell transplantation (HSCT) in children with an incidence of 17%–52% [1, 2, 3, 4, 5]. It can lead to visceral dissemination that has the potential to be fatal, bacterial superinfection, postherpetic neuralgia resulting in quality of life impairment, and long‐term sequelae. Various risk factors for VZV reactivation have been identified, including graft‐versus‐host disease (GVHD), delayed immune reconstitution, age over 10 years old, and total body irradiation‐based conditioning regimen (TBI) [4, 5, 6]. While the literature demonstrates acyclovir's effectiveness against VZV reactivation disease after allogeneic HSCT, there is no consensus on the optimal dosage and duration of treatment [7, 8, 9, 10, 11, 12, 13, 14]. In 2009, European guidelines recommended a 1‐year prophylaxis with oral acyclovir for VZV‐seropositive allogeneic HSCT recipients, which may be extended in the presence of GVHD and immunosuppressive therapy [14]. Some clinicians advocate continuing prophylaxis until 6 months or even less after cessation of immunosuppression [7, 11]. However, the optimal duration of prophylaxis and the risk factors for VZV reactivation after cessation of prophylaxis remain unclear. In our center, Lille university hospital, France, we used the absolute count of CD4+ cells in lymphocytes immunophenotyping to determine when to discontinue acyclovir prophylaxis, specifically when this count exceeds 0.2 × 10^9^/L. Despite this practice, we have observed VZV reactivation in some patients. Lymphocytes immunophenotyping is a simple blood test, useful to evaluate regeneration of immunity and we wonder which criteria other than the absolute rate of CD4 would help to decide when to stop acyclovir prophylaxis.

The purpose of this retrospective study was to investigate whether lymphocytes immunophenotyping was an efficient tool for determining the optimal duration of acyclovir prophylaxis following allogeneic HSCT in children. Additional goals were to evaluate the incidence, clinical characteristics, and risk factors for VZV infections after HSCT.

## 2 Patients and Methods

### 2.1 Patient Population

We retrospectively analyzed, from January 2007 to December 2011, all 90 patients aged less than 18 years who underwent an allogeneic HSCT at the referral center of Lille University Hospital, France. All patients' follow‐ups were performed in our center until May 2015. All procedures performed in this study involving human participants were in accordance with the ethical standards of the institutional and/or national research committee and with the 1964 Helsinki declaration and its later amendments or comparable ethical standards. Informed consent was obtained from all participants included in the study.

### 2.2 Transplantation Procedure

Myeloablative conditioning regimen consisted of 12 Grays fractionated TBI combined with etoposide (60 mg/kg) or busulfan (3.2–4.8 mg/kg/day for 4 days) combined with cyclophosphamide (200 mg/kg). Rabbit antithymocyte globulin ranged from 5 to 20 mg/kg.

Prophylaxis for GVHD consisted of cyclosporine alone (2 mg/kg intravenous twice daily) or combined with short‐term methotrexate (10 mg/m^2^ intravenous on days +1, +2, +3, and +6), mycophenolate mofetil (600 mg/m^2^ twice daily) or prednisolone. Acute and chronic GVHD (aGVHD and cGVHD) were defined by standard criteria [15, 16].

Immunoglobulin substitution was given to patients who underwent cord blood transplantation until 6 months posttransplantation or to patients with an immunoglobulin G level under 6 g/L.

### 2.3 VZV Prophylaxis

According to European guidelines, patients who had a positive immunoglobulin G antibody titer to herpes simplex virus or VZV, as determined by enzyme‐linked immunoabsorbent assay, received prophylaxis with intravenous acyclovir 250 mg/m^2^ three times per day from day one until engraftment; followed by 40 mg/kg orally twice a day or valaciclovir 500 mg once (weight < 40 kg) or 500 mg twice daily (weight > 40 kg) [14]. Acyclovir dose adjustment was performed only in case of severe renal failure (creatinine clearance < 10 mL/min/1.73 m^2^). This prophylaxis was continued until the CD4+ lymphocytes count exceeded 0.2 × 10^9^/L after cessation of immunosuppression.

### 2.4 Definitions [1]

Herpes zoster was defined by the appearance of characteristic vesicular skin lesions on an erythematous base within a dermatome. Relapsed disease was defined as reappearance of classic lesions within 2 months after the first episode. Recurrent infection was defined as reappearance of classic lesions more than 2 months after the initial episode. Cutaneous dissemination of herpes zoster was defined as the subsequent appearance of lesions in noncontiguous dermatomes. Varicella was defined as a generalized eruption without dermatomal localization. Visceral organ dissemination was defined as histological or clinical evidence of organ involvement in the absence of other identified etiology. Postherpetic neuralgia was defined as the need to provide ongoing pain medications to treat pain in a dermatomal distribution following the resolution of skin lesions.

### 2.5 Assessment of Immune Reconstitution

Immune reconstitution was monitored through lymphocytes immunophenotyping performed every 3 months until discontinuation of prophylaxis with cotrimoxazole and acyclovir. Lymphocytes immunophenotyping was analyzed in peripheral blood by flow cytometry.

### 2.6 Statistical Analysis

Data are presented as counts and percentages for categorical variables, and mean ± SD or median (range) for continuous variables depending on normality. Normality of distribution was checked graphically and by using the Shapiro–Wilk test.

The cumulative incidence of VZV infection from HSCT was determined by using the Kalbfleisch and Prentice method by treating mortality as competing events. Among the 77 patients with prophylaxis, we assessed the association of cessation of prophylaxis on VZV infection by using cause‐specific Cox proportional regression hazard model, treating cessation of prophylaxis as a time‐varying variable. In the overall study sample (*n* = 84), we assessed in univariate analyses, the association of patient's characteristics and posttransplant VZV infection by estimating the hazard of VZV infection occurring with permutations of each patient's characteristics by using univariable cause‐specific Cox proportional hazard regression models. Proportional hazard assumptions were assessed using Schoenfeld residual plots and no deviation was observed. Characteristics associated with posttransplant VZV infection in univariate analyses (at *p* < 0.20 significance level, except cord blood graft colinear with immunoglobulin substitution), were considered as candidates variables to a backward‐stepwise multivariable cause‐specific Cox proportional hazard regression model using Firth's penalized likelihood method to account the small number of events. Finally, among the subset of 42 patients with available data on lymphocytes immunophenotyping before the cessation of anti‐VZV prophylaxis, we assessed the hazard of various lymphocyte count/ratio thresholds (thresholds determined by the median value for all lymphocyte measures) on subsequent VZV infection after cessation of anti‐VZV prophylaxis using univariable cause‐specific Cox proportional regression hazard models. Data were analyzed using SAS software package, release 9.4 (SAS Institute, Cary, NC, USA). Statistical testing was done at the two‐tailed *α* level of 0.05.

## 3 Results

### 3.1 Transplantation Outcomes

**FIGURE 1:**
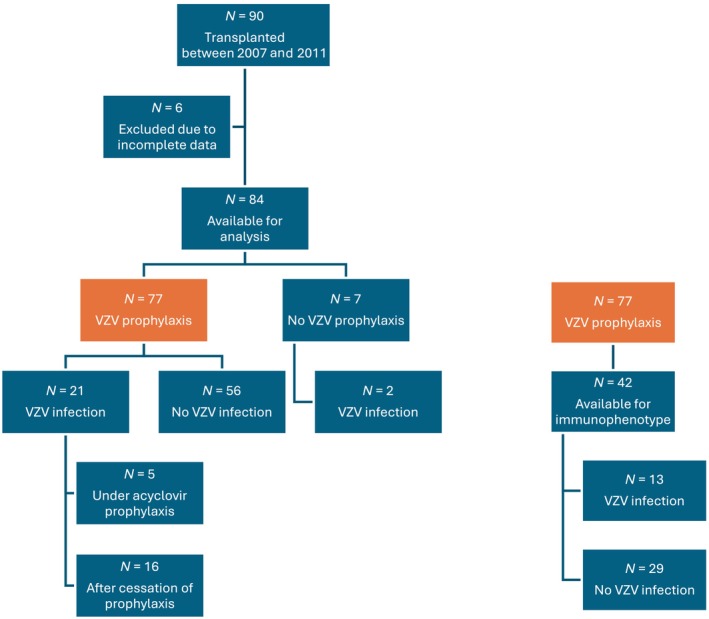
Flowchart of enrolled patients. VZV, varicella‐zoster virus.

Among the 90 patients who underwent HSCT, six were excluded because of a lack of data (more than 50% of data was missing, Figure 1). The clinical characteristics of the 84 patients included are displayed in Table 1. The median follow‐up after HSCT was 4.6 years (range, 42 days–8.2 years). All patients but one experienced neutrophil engraftment. The median duration to neutrophil recovery > 500/mm^3^ was 22 days (range, 9–60). Grade II–IV aGVHD was observed in 33 patients (40.5%), and 17.8% had cGVHD. The duration of the immunosuppressive therapy ranged from 42 days to 2.2 years after HSCT (median duration 5 months). Twenty‐nine patients had a viral infection other than VZV (cytomegalovirus, Epstein Barr virus, human herpesvirus 6 or adenovirus infections) which occurred from 1 day to 2.9 years after HSCT (median 38 days, incidence rate 1.42 per 100 patients‐months; exact 95% CI, 0.92–2.09). Twenty‐eight patients (32%) died during the follow‐up after HSCT including 9 (11%) of nonrelapse mortality.

**TABLE 1:** Patient characteristics.

	Total = 84
Age, years, median (range)	10 (0.3–19.6)
Male, *n* (%)	45 (54)
Underlying disease, *n* (%)	
Hematological malignancy	63 (75)
Neuroblastoma	2 (2.4)
Nonmalignant disease	19 (22.6)
Malignancy status at time of transplant, *n* (%)	
First complete remission	31 (49.2)
Others	32 (50.8)
Graft source, *n* (%)	
Bone marrow	51 (60.7)
Cord blood	29 (34.5)
Cord blood + bone marrow	3 (3.6)
Peripheral blood stem cells	1 (1.2)
Donor type, *n* (%)	
Matched related	33 (39.3)
Haploidentical	2 (2.4)
Matched unrelated	28 (33.3)
Mismatched unrelated	21 (25)
Conditioning regimen, *n* (%)	
Myeloablative/nonmyeloablative	74/10 (88/12)
TBI‐based	41 (48.8)
Chemo‐based	43 (51.2)
Antithymocyte globulin use, *n* (%)	39 (46.4)
GVHD prophylaxis, *n* (%)	
Cyclosporine + methotrexate	27 (32.1)
Cyclosporine + mycophenolate mofetil	16 (19)
Cyclosporine + prednisolone	14 (16.7)
Cyclosporine	24 (28.6)
No immunosuppressive treatment	3 (3.6)
Positive VZV recipient serology, *n* (%)	74 (88)
Immunoglobulin substitution, *n* (%)	32 (38)
Acute GVHD (grade II–IV)	33 (40.5)
Chronic GVHD	15 (17.8)

Abbreviations: GVHD, graft‐versus‐host disease; TBI, total body irradiation; VZV, varicella‐zoster virus.

### 3.2 Incidence and Clinical Features of VZV Infections

Twenty‐three of 84 patients (27%) developed VZV infection (Figure 1) at a median of 7.2 months after HSCT (range, 3 days–3.6 years), given an incidence rate of 1.28 per 100 patients‐months (exact 95% CI, 0.80–1.92). VZV infections characteristics are shown in Table 2. Eight of the 23 patients who had VZV infection (34.8%) needed hospitalization to receive intravenous acyclovir. Two patients had a cerebral stroke after VZV infection. The first one, 6 months after cord blood transplantation, had a right herpes zoster ophtalmicus followed by a myocarditis at 3 weeks of zoster then an arterial ischemia of left internal capsule with contralateral hemiparesis at 6 weeks of zoster. The resulting ptosis and heart insufficiency persisted. The second patient, 8 months after marrow transplantation, had left herpes zoster ophtalmicus followed by ischemic left middle cerebral artery stroke 10 months after zoster and consequently developed sequelae ptosis. For those two patients with cerebral stroke, no other etiology was identified.

**TABLE 2:** Clinical outcomes of VZV disease.

	Total = 23
Presentation of vesicular eruption	
Localized zoster, *n* (%)	18 (78.3)
Thoracic	5
Lumbar, sacral	6
Facial	4
Cervical	1
Arm	2
Skin dissemination, *n* (%)	1 (4.3)
Varicella, *n* (%)	2 (8.7)
Unknown, *n* (%)	2 (8.7)
Relapsed disease, *n* (%)	2 (8.7)
Recurrent infection, *n* (%)	1 (4.3)
Hospitalized, *n* (%)	8 (34.8)
Complications, *n* (%)	
Postherpetic neuralgia	2 (8.7)
Sequella ptosis	2 (8.7)
Visceral dissemination	2 (8.7)

Abbreviation: VZV, varicella‐zoster virus.

**FIGURE 2:**
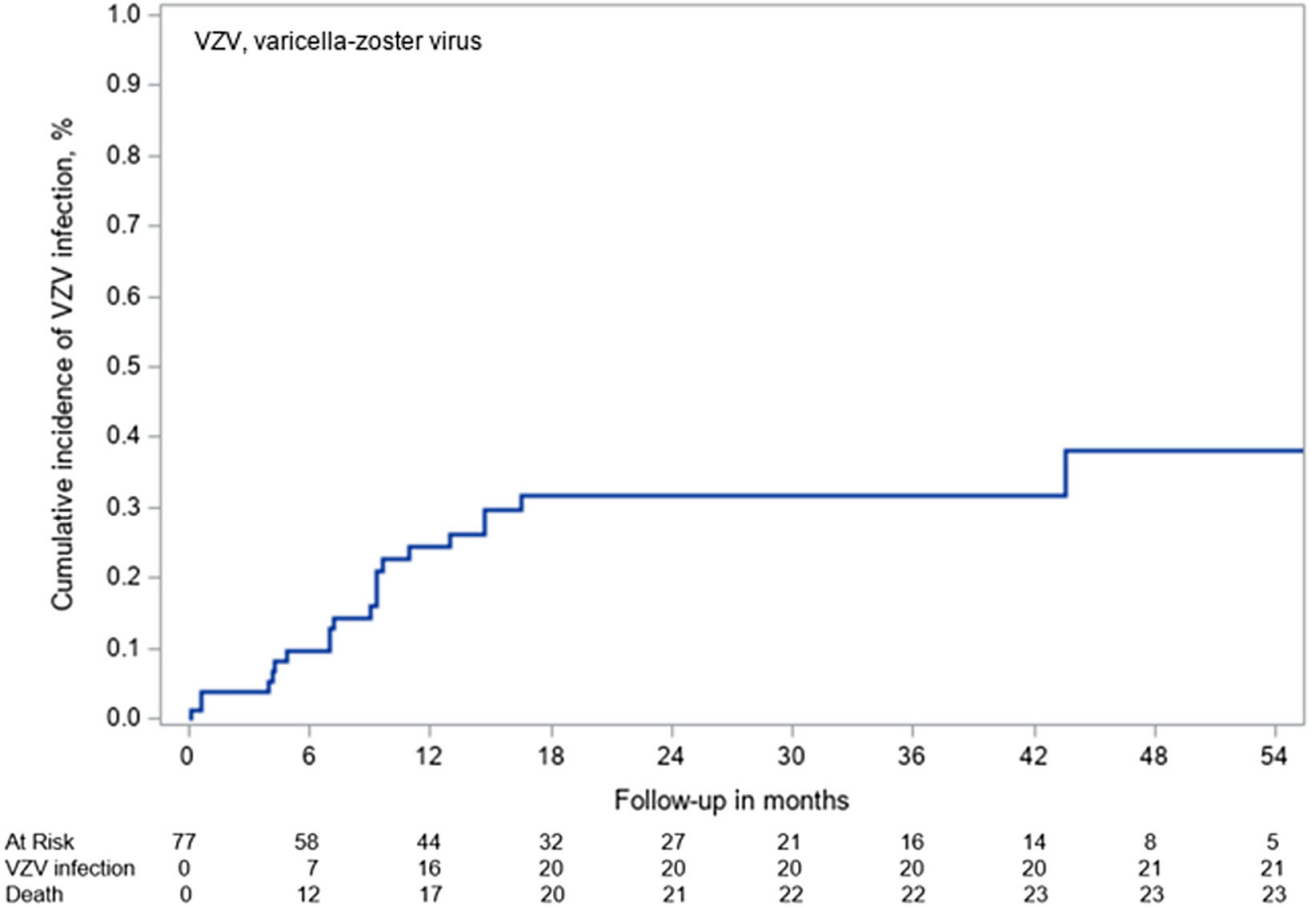
Cumulative incidence of VZV infection based on prophylaxis.

Among the 77 patients who received VZV prophylaxis (according to our practical guidelines), 21 developed VZV infection (Figures 1 and 2) with an incidence rate of 1.30 per 100 patients‐months (exact 95% CI, 0.81–2.01). The two patients with VZV infection who did not receive acyclovir prophylaxis were transplanted with a cord blood unit: one patient underwent an HSCT for severe combined immunodeficiency. He had a mild varicella at age 4 months but was seronegative when he was 2 years old just before transplant; the second patient was seronegative before transplant and developed primary varicella at 5 months posttransplant after exposure to a person with active VZV infection. The median duration of prophylaxis was the same for patients who presented a posttransplant VZV infection than for those without VZV infection, 8.4 months (range, 3–11.4 months) versus 8.7 months (range, 3.7–16.2 months). Of the 21 VZV infections, only five occurred under prophylaxis, from 3 to 19 days after HSCT for three patients, of whom one had a proven acyclovir resistance, and at day 130 and day 147 for two patients due to poor compliance. The higher risk for VZV reactivation after stopping prophylaxis was significant with a cause‐specific hazard ratio (cHR) of 23.2 (95% CI, 2.72–197.4, *p* = 0.04). The remaining 16 VZV infections (Figure 1) occurred after cessation of prophylaxis within a median of 49 days (range, 11 days–5.8 months).

### 3.3 Risk Factors for VZV Infections

**FIGURE 3:**
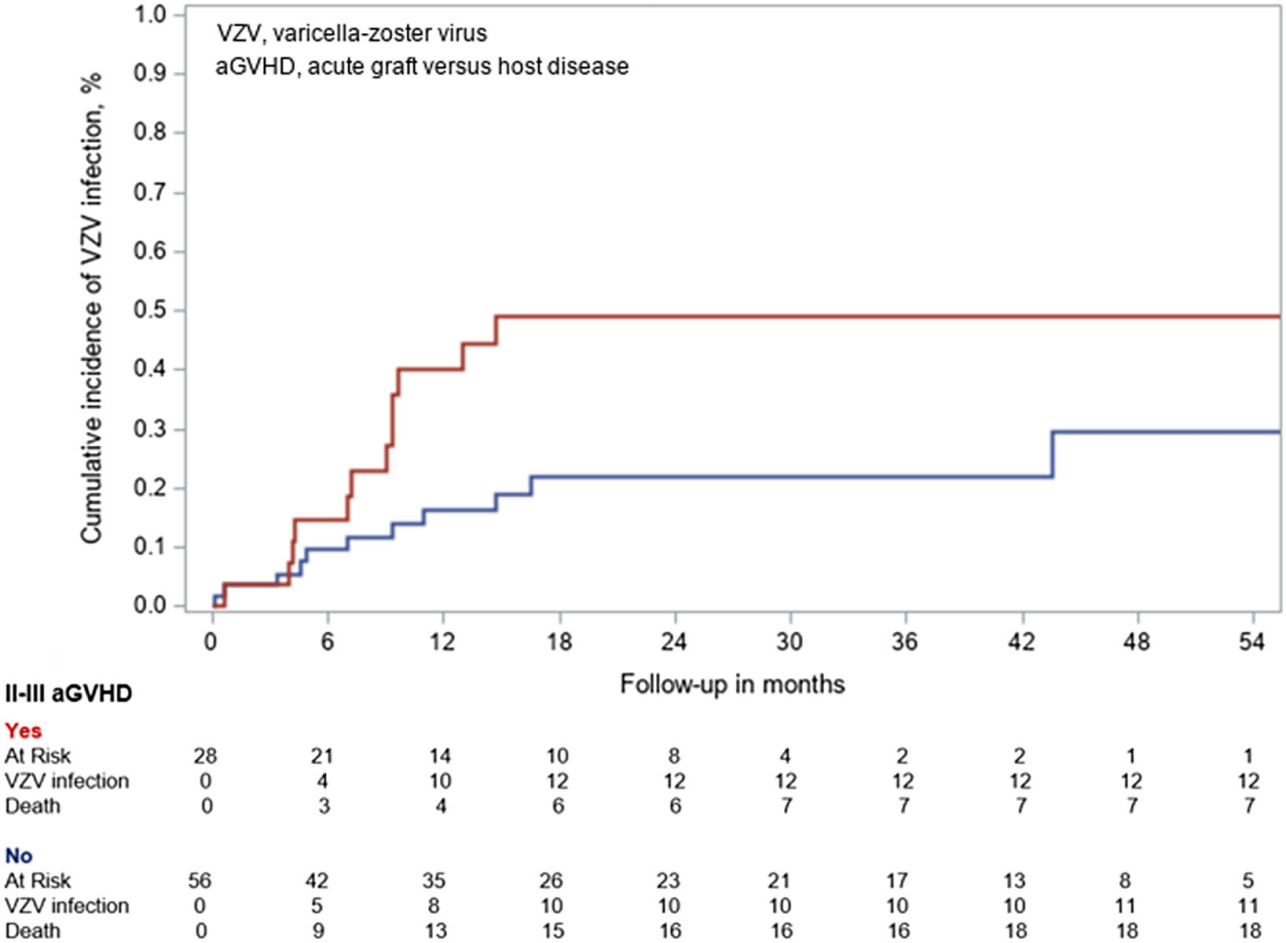
Cumulative incidence of VZV infection based on II–III aGVHD.

Table 3 shows the univariate associations of risk factors for VZV infections among the overall study population (*n* = 84). TBI‐based conditioning regimen, immunoglobulin substitution, cord blood transplant, and antithymocyte globulin were associated with a higher risk for VZV infection, although the association with anti‐thymocyte globulin did not reach the significance level (cHR 2.05, 95% CI, 0.88–4.75). II–III aGVHD also appeared to be a risk factor for VZV infection (cHR 2.43 [95% CI, 1.07–5.53]; *p* = 0.03) (Figure 3). In the multivariate analysis, including candidates' factors associated with a *p* < 0.20 in univariate analysis (except cord blood transplant colinear with immunoglobulin substitution), conditioning regimen with TBI, immunoglobulin substitution and antithymocyte globulin were independently associated with higher VZV infection (Table 3).

**TABLE 3:** Risk factors for VZV infection after transplantation.

				Total = 84		
	Univariate analysis			Multivariate analysis		
Potential risk factors	HR	95% CI	*p*	HR^a^	95% CI	*p*
Median age at diagnosis ≥ 10 years	0.49	0.33–1.71	0.49			
Recipient negative VZV serology	0.23	0.03–1.71	0.15			
Donor negative VZV serology	1.50	0.65–3.48	0.34			
First complete remission	0.73	0.28–1.93	0.53			
Cord blood graft	2.6	1.10–5.90	0.03			
Sibling donor	0.75	0.36–1.71	0.49			
HLA compatibility			0.14			
10/10 or 6/6	0.37	0.14–0.99	0.05			
9/10 or 4–5/6	0.42	0.10–1.7	0.22			
Nonmyeloablative conditioning	1.40	0.47–4.13	0.54			
Conditioning with TBI	3.17	1.29–7.83	0.01	4.15	1.52–11.30	0.006
Antithymocyte globulin use	2.05	0.88–4.75	0.09	3.60	1.45–8.94	0.006
Immunoglobulin substitution	3.24	1.41–7.44	0.006	2.53	1.06–6.02	0.037
GVHD	0.88	0.37–2.07	0.77			
Acute GVHD (grade II–III)	2.43	1.07–5.53	0.03			
Chronic GVHD	1.88	0.56–6.34	0.31			

Abbreviations: GVHD, graft‐versus‐host disease; TBI, total body irradiation; VZV, varicella‐zoster virus.

^a^ Hazard ratio calculated in backward‐stepwise multivariable cause‐specific Cox's proportional hazard model with Firth's penalized likelihood method including the following candidate variables: conditioning with total body irradiation, immunoglobulin substitution, acute GVH II–III, and antithymocyte globulin.

### 3.4 Lymphocytes Immunophenotyping Before Prophylaxis Cessation

The lymphocytes immunophenotyping assessed before prophylaxis cessation were available for 42 patients. The median duration between lymphocyte immunophenotyping and prophylaxis cessation was 20.5 days (range, 0–92 days). Among the 42 patients, 13 had VZV infection after prophylaxis cessation (Figure 1). The distributions of last available lymphocytes immunophenotyping before prophylaxis cessation according to occurrence or not of VZV infection during follow‐up after cessation were reported in Supporting Information. The risk of VZV infection was not significantly increased under the median (absolute rate or percentage) value for CD4+ or CD19+ lymphocytes and under the median value of CD8+ and CD19+ percentages (Table 4). The risk of VZV infection was significantly increased for patients with percentage of CD4+ lymphocytes < 23% (cHR 3.28 [95% CI, 1.09–9.81]; *p* = 0.03) or for patients with CD4^+^/CD8^+^ ratio under 0.9 (cHR 3.13 [95% CI, 1.04–9.36]; *p* = 0.04). The result suggested a potential association between a low rate of CD8+ lymphocytes and an increased risk of VZV infection (cHR 2.49 [95% CI, 0.77–8.08]). However, this association was not statistically significant.

**TABLE 4:** Association of lymphocytes immunophenotype performed before discontinuing anti‐VZV prophylaxis with the occurrence of VZV infection after discontinuing prophylaxis.

			Total = 42
Lymphocytes	VZV infection, *n*	Incidence per 100 patient‐months (95% CI)	cHR^a^ (95% CI)
Total lymphocyte (10^9^/L)			
< 1454	8	1.33 (0.57–2.63)	1.70 (0.56–5.20)
≥ 1454	5	1.01 (0.32–2.36)	1.00 (ref.)
CD3%			
< 52	7	1.33 (0.53–2.73)	1.38 (0.46–4.12)
≥ 51	6	1.01 (0.38–2.31)	1.00 (ref.)
CD3 absolute value (10^9^/L)			
< 805	7	1.13 (0.45–2.33)	1.23 (0.41–3.67)
≥ 805	6	1.26 (0.46–2.75)	1.00 (ref.)
CD4%			
< 23	8	2.23 (0.97–4.45)	3.28 (1.09–9.81)
≥ 23	5	0.67 (0.21–1.58)	1.00 (ref.)
CD4 absolute value (10^9^/L)			
< 374	7	1.12 (0.46–2.25)	1.27 (0.43–3.80)
≥ 374	6	1.12 (0.44–2.64)	1.00 (ref.)
CD8%			
< 29	7	1.13 (0.51–2.65)	1.14 (0.38–3.40)
≥ 29	6	1.11 (0.40–2.38)	1.00 (ref.)
CD8 absolute value (10^9^/L)			
< 376	9	1.13 (0.52–2.67)	2.49 (0.77–8.08)
≥ 376	4	1.11 (0.39–2.34)	1.00 (ref.)
CD4/CD8			
< 0.9	8	2.25 (1.11–4.49)	3.13 (1.04–9.36)
≥ 0.9	5	0.65 (0.21–1.52)	1.00 (ref.)
CD19%			
< 27	6	1.11 (0.38–2.24)	0.76 (0.25–2.25)
≥ 27	7	1.32 (0.53–2.28)	1.00 (ref.)
CD19 absolute value (10^9^/L)			
< 331	8	1.14 (0.62–2.85)	1.62 (0.53–4.96)
≥ 331	5	0.92 (0.29–0.22)	1.00 (ref.)

Abbreviation: VZV, varicella‐zoster virus.

^a^ cHR indicates cause‐specific hazard ration associated with lower lymphocytes values (according to the median threshold value).

## 4 Discussion

In allogeneic HSCT settings, acyclovir is highly effective in preventing VZV reactivation disease during the time of its administration. However, reactivation can occur after discontinuation of prophylaxis [17, 18, 19, 20, 21]. In this retrospective study of children receiving allogeneic HSCT, the incidence of VZV infection was 27%, in which 35% of them were hospitalized. Nine percent of the infected patients (two patients) experienced postherpetic neuralgia. Nine percent experienced sequelae ptosis, which is a known complication of ophthalmic zoster [22, 23]. Nine percent experienced visceral dissemination with ischemic strokes probably secondary to postzoster angiopathy as described in literature [24]. One of those two patients with visceral dissemination also had a myocarditis. In Berman's pediatric study, among 201 allogeneic HSCT, the incidence of VZV infection was 45% at 5 years with 29% of postherpetic neuralgia and 7% of visceral dissemination [1]. In Leung's pediatric study, among 109 auto and allogeneic HSCT, incidence was 31% with 11% of visceral dissemination [5]. Both studies had a shorter virus infection prophylaxis with 1–4 months of ganciclovir for cytomegalovirus prophylaxis and 1 month of acyclovir prophylaxis for herpes simplex virus. In our study, despite the necessity of a CD4+ lymphocytes count exceeding 0.2 × 10^9^/L and cessation of immunosuppression to withdrawal acyclovir, we still observed VZV reactivation and its morbidity.

Several retrospective pediatric and adult studies have indicated that the following: patients older than 10 years, underlying hematological malignancies, conditioning regimen with TBI, cord blood transplant, GVHD, use of antithymocyte globulin, and delayed immune reconstitution were risk factors for VZV reactivation after auto or allogeneic HSCT [4, 5, 6, 7, 25]. In an adult prospective study, Boeckh et al. showed that the most important risk factors for late VZV disease were continued immunosuppression and related donor status [21]. We confirmed that use of antithymocyte globulin, conditioning regimen with TBI, and immunoglobulin substitution were risk factors for VZV infection.

CD4 T cell immunity is critical for the control of VZV reactivation [26, 27, 28, 29]. Studying regeneration of immunity and VZV infection after high‐dose chemotherapy and HSCT autografts in children, Takaue et al. showed that the CD4/CD8 ratio markedly decreased, with a nadir at 3 months, due to both abnormally low levels of CD4+ cells and sustained higher levels of CD8+ lymphocytes. These abnormalities were sustained for more than 12 months postgraft [30]. A study from Aytaç et al. also observed that post HSCT VZV infection was associated with lower CD4/CD8 ratios in children [31].

We tried to establish a correlation between the last available lymphocytes immunophenotyping before prophylaxis cessation and VZV infections. In our study, the incidence of VZV infections increased significantly when the percentage of CD4+ lymphocytes was < 23% or when the ratio of CD4+/CD8+ was under 0.9 before prophylaxis cessation. Due to the small number of patients with available data on lymphocytes immunophenotyping at the time of prophylaxis cessation, multivariate analysis was not used to assess the independent association of lymphocytes immunophenotyping on VZV infection.

The findings of our study are derived from observational retrospective analysis of limited sample size in which certain limitations cannot be ruled out—selection bias, classification bias, and confounding bias. Some associations may have been overlooked due to the sample size. Furthermore, we cannot exclude some false positive findings, especially in the context of multiple testing issue. The present findings should be only considered as hypothesis generating and should be replicated in further larger prospective studies.

To conclude, in the absence of a test to measure VZV‐specific cell‐mediated immunity, adopting an approach based on lymphocytes immunophenotype with the percentage of CD4+ lymphocytes and the ratio CD4+/CD8+, may offer a biologically relevant approach for discontinuing acyclovir prophylaxis.

## Author Contributions

**Eva de Berranger**: formal analysis, writing – original draft, investigation. **Anne‐Flore Derache**: formal analysis, writing – original draft, investigation. **Nassima Ramdane**: data curation, formal analysis. **Julien Labreuche**: data curation, formal analysis. **Pauline Navarin**: resources. **Fanny Gonzales**: resources. **Wadih Abou‐Chahla**: resources. **Brigitte Nelken**: resources. **Bénédicte Bruno**: conceptualization, project administration, writing – review and editing, supervision.

## Ethics Statement

All procedures performed in this study involving human participants were in accordance with the ethical standards of the institutional and/or national research committee and with the 1964 Helsinki declaration and its later amendments or comparable ethical standards.

## Consent

Informed consent was obtained from all participants included in the study.

## Conflicts of Interest

The authors declare no conflicts of interest.

## Supporting information


**Data S1.** Boxplots of lymphocytes immunophenotype values assessed before discontinuing prophylaxis.

## Data Availability

The data that support the findings of this study are available on request from the corresponding author. The data are not publicly available due to privacy or ethical restrictions.

## References

[1] J. N. Berman , M. Wang , W. Berry , D. S. Neuberg , and E. C. Guinan , “Herpes Zoster Infection in the Post‐Hematopoietic Stem Cell Transplant Pediatric Population May Be Preceded by Transaminitis: An Institutional Experience,” Bone Marrow Transplantation 37, no. 1 (January 2006): 73–80. 16247423 10.1038/sj.bmt.1705191

[2] N. Doki , S. Miyawaki , M. Tanaka , et al., “Visceral Varicella Zoster Virus Infection After Allogeneic Stem Cell Transplantation,” Transplant Infectious Disease 15, no. 3 (June 2013): 314–318. 23551634 10.1111/tid.12073

[3] H. Kawasaki , J. Takayama , and M. Ohira , “Herpes Zoster Infection After Bone Marrow Transplantation in Children,” Journal of Pediatrics 128, no. 3 (March 1996): 353–356. 8774503 10.1016/s0022-3476(96)70280-9

[4] Y. Koc , K. B. Miller , D. P. Schenkein , et al., “Varicella Zoster Virus Infections Following Allogeneic Bone Marrow Transplantation: Frequency, Risk Factors, and Clinical Outcome,” Biology of Blood and Marrow Transplantation 6, no. 1 (2000): 44–49. 10707998 10.1016/s1083-8791(00)70051-6

[5] T. F. Leung , K. W. Chik , C. K. Li , et al., “Incidence, Risk Factors and Outcome of Varicella‐Zoster Virus Infection in Children After Haematopoietic Stem Cell Transplantation,” Bone Marrow Transplantation 25, no. 2 (January 2000): 167–172. 10673675 10.1038/sj.bmt.1702119

[6] C. L. Vermont , E. C. M. Jol‐van der Zijde , P. Hissink Muller , et al., “Varicella Zoster Reactivation After Hematopoietic Stem Cell Transplant in Children Is Strongly Correlated With Leukemia Treatment and Suppression of Host T‐Lymphocyte Immunity,” Transplant Infectious Disease 16, no. 2 (April 2014): 188–194. 24438482 10.1111/tid.12180

[7] S. B. Han , S. K. Kim , J. W. Lee , et al., “Varicella Zoster Virus Infection After Allogeneic Hematopoietic Cell Transplantation in Children Using a Relatively Short Duration of Acyclovir Prophylaxis: A Retrospective Study,” Medicine (Baltimore) 96, no. 14 (April 2017): e6546. 28383421 10.1097/MD.0000000000006546 PMC5411205

[8] Y. Tatebe , S. Ushio , S. Esumi , et al., “Low‐Dose Acyclovir for Prophylaxis of Varicella‐Zoster Virus Reactivation After Hematopoietic Stem Cell Transplantation in Children,” Pediatric Blood & Cancer 69, no. 12 (December 2022): e29979. 36151963 10.1002/pbc.29979

[9] D. H. Kim , H. Messner , M. Minden , et al., “Factors Influencing Varicella Zoster Virus Infection After Allogeneic Peripheral Blood Stem Cell Transplantation: Low‐Dose Acyclovir Prophylaxis and Pre‐Transplant Diagnosis of Lymphoproliferative Disorders,” Transplant Infectious Disease 10, no. 2 (April 2008): 90–98. 17605742 10.1111/j.1399-3062.2007.00247.x

[10] K. Oshima , T. Takahashi , T. Mori , et al., “One‐Year low‐Dose Valacyclovir as Prophylaxis for Varicella Zoster Virus Disease After Allogeneic Hematopoietic Stem Cell Transplantation. A Prospective Study of the Japan Hematology and Oncology Clinical Study Group,” Transplant Infectious Disease 12, no. 5 (October 2010): 421–427. 20626711 10.1111/j.1399-3062.2010.00541.x

[11] V. R. Wormser , N. I. Agudelo Higuita , R. Ramaswami , and D. P. Melendez , “Hematopoietic Stem Cell Transplantation and the Noncytomegalovirus Herpesviruses,” Transplant Infectious Disease 25, no. Suppl 1 (November 2023): e14201. 38041493 10.1111/tid.14201

[12] Y. Wada‐Shimosato , R. Tanoshima , K. Hiratoko , et al., “Effectiveness of Acyclovir Prophylaxis Against Varicella Zoster Virus Disease After Allogeneic Hematopoietic Cell Transplantation: A Systematic Review and Meta‐Analysis,” Transplant Infectious Disease 21, no. 3 (June 2019): e13061. 30756465 10.1111/tid.13061

[13] M. Ifversen , R. Meisel , P. Sedlacek , et al., “Supportive Care During Pediatric Hematopoietic Stem Cell Transplantation: Prevention of Infections. A Report From Workshops on Supportive Care of the Paediatric Diseases Working Party (PDWP) of the European Society for Blood and Marrow Transplantation (EBMT),” Frontiers in Pediatrics 9 (2021): 705179. 34395344 10.3389/fped.2021.705179 PMC8358428

[14] J. Styczynski , P. Reusser , H. Einsele , et al., “Management of HSV, VZV and EBV Infections in Patients With Hematological Malignancies and After SCT: Guidelines From the Second European Conference on Infections in Leukemia,” Bone Marrow Transplantation 43, no. 10 (May 2009): 757–770. 19043458 10.1038/bmt.2008.386

[15] A. H. Filipovich , D. Weisdorf , S. Pavletic , et al., “National Institutes of Health Consensus Development Project on Criteria for Clinical Trials in Chronic Graft‐Versus‐Host Disease: I. Diagnosis and Staging Working Group Report,” Biology of Blood and Marrow Transplantation 11, no. 12 (December 2005): 945–956. 16338616 10.1016/j.bbmt.2005.09.004

[16] D. Przepiorka , D. Weisdorf , P. Martin , et al., “1994 Consensus Conference on Acute GVHD Grading,” Bone Marrow Transplantation 15, no. 6 (June 1995): 825–828. 7581076

[17] O. Blennow , G. Fjaertoft , J. Winiarski , P. Ljungman , J. Mattsson , and M. Remberger , “Varicella‐Zoster Reactivation After Allogeneic Stem Cell Transplantation Without Routine Prophylaxis—The Incidence Remains High,” Biology of Blood and Marrow Transplantation 20, no. 10 (October 2014): 1646–1649. 24914821 10.1016/j.bbmt.2014.06.002

[18] V. Erard , K. A. Guthrie , C. Varley , et al., “One‐Year Acyclovir Prophylaxis for Preventing Varicella‐Zoster Virus Disease After Hematopoietic Cell Transplantation: No Evidence of Rebound Varicella‐Zoster Virus Disease After Drug Discontinuation,” Blood 110, no. 8 (October 2007): 3071–3077. 17515400 10.1182/blood-2007-03-077644

[19] K. Kawamura , H. Wada , R. Yamasaki , et al., “Prophylactic Role of Long‐Term Ultra‐Low‐Dose Acyclovir for Varicella Zoster Virus Disease After Allogeneic Hematopoietic Stem Cell Transplantation,” International Journal of Infectious Diseases 19 (February 2014): 26–32. 24211377 10.1016/j.ijid.2013.09.020

[20] K. J. Thomson , D. P. Hart , L. Banerjee , K. N. Ward , K. S. Peggs , and S. Mackinnon , “The Effect of Low‐Dose Aciclovir on Reactivation of Varicella Zoster Virus After Allogeneic Haemopoietic Stem Cell Transplantation,” Bone Marrow Transplantation 35, no. 11 (June 2005): 1065–1069. 15806119 10.1038/sj.bmt.1704959

[21] M. Boeckh , H. W. Kim , M. E. D. Flowers , J. D. Meyers , and R. A. Bowden , “Long‐Term Acyclovir for Prevention of Varicella Zoster Virus Disease After Allogeneic Hematopoietic Cell Transplantation—A Randomized Double‐Blind Placebo‐Controlled Study,” Blood 107, no. 5 (March 2006): 1800–1805. 16282339 10.1182/blood-2005-09-3624 PMC1895699

[22] R. C. Walton and K. L. Reed , “Herpes Zoster Ophthalmicus Following Bone Marrow Transplantation in Children,” Bone Marrow Transplantation 23, no. 12 (June 1999): 1317–1320. 10414922 10.1038/sj.bmt.1701796

[23] B. P. Yawn , P. C. Wollan , J. L. St Sauver , and L. C. Butterfield , “Herpes Zoster eye Complications: Rates and Trends,” Mayo Clinic Proceedings 88, no. 6 (June 2013): 562–570. 23664666 10.1016/j.mayocp.2013.03.014 PMC3788821

[24] C. Amlie‐Lefond and D. Gilden , “Varicella Zoster Virus: A Common Cause of Stroke in Children and Adults,” Journal of Stroke and Cerebrovascular Diseases 25, no. 7 (July 2016): 1561–1569. 27138380 10.1016/j.jstrokecerebrovasdis.2016.03.052 PMC4912415

[25] C. B. Steer , J. Szer , J. Sasadeusz , J. P. Matthews , J. A. Beresford , and A. Grigg , “Varicella‐Zoster Infection After Allogeneic Bone Marrow Transplantation: Incidence, Risk Factors and Prevention With Low‐Dose Aciclovir and Ganciclovir,” Bone Marrow Transplantation 25, no. 6 (March 2000): 657–664. 10734301 10.1038/sj.bmt.1702190

[26] K. Haberthur , F. Engelmann , B. Park , et al., “CD4 T Cell Immunity Is Critical for the Control of Simian Varicella Virus Infection in a Nonhuman Primate Model of VZV Infection,” PLoS Pathogens 7, no. 11 (November 2011): e1002367. 22102814 10.1371/journal.ppat.1002367 PMC3213099

[27] J. Ogonek , M. Kralj Juric , S. Ghimire , et al., “Immune Reconstitution After Allogeneic Hematopoietic Stem Cell Transplantation,” Frontiers in Immunology 7 (2016): 507. 27909435 10.3389/fimmu.2016.00507 PMC5112259

[28] A. E. Edwards , C. Suarez , and J. Lambourne , “Lesson of the Month: Late, Disseminated Herpes Zoster Reactivation in a Stem Cell Transplant Recipient: Implications for Post‐Transplant Prophylaxis and Immune Monitoring,” Clinical Medicine 21, no. 3 (May 2021): e309–e312. 34001587 10.7861/clinmed.2021-0064 PMC8140707

[29] M. R. Heldman , K. M. Aagaard , and J. A. Hill , “Assessing and Restoring Adaptive Immunity to HSV, VZV and HHV‐6 in Solid Organ and Hematopoietic Cell Transplant Recipients,” Clinical Microbiology and Infection 28, no. 10 (October 2022): 1345–1350. 35150885 10.1016/j.cmi.2022.02.001 PMC9363517

[30] Y. Takaue , Y. Okamoto , Y. Kawano , et al., “Regeneration of Immunity and Varicella‐Zoster Virus Infection After High‐Dose Chemotherapy and Peripheral Blood Stem Cell Autografts in Children,” Bone Marrow Transplantation 14, no. 2 (August 1994): 219–223. 7994235

[31] S. Aytaç , S. S. Yalçin , O. Küçükbayrak , M. Çetın , and D. Uçkan , “Dynamics in Children and Adolescents Who Experience Varicella Zoster Virus Infections After Haematopoietic Stem Cell Transplantation: A Case‐Control Study,” Epidemiology and Infection 139, no. 11 (November 2011): 1701–1709. 21226986 10.1017/S0950268810003031

