# Supplementary figures and images for: VZV Prophylaxis After Allogeneic Hematopoietic Stem Cell Transplantation in Children: When to Stop?
Source: Cancer Rep (Hoboken). 2024 Nov 7;7(11):e70015. doi: 10.1002/cnr2.70015 (PMC11541057; doi:10.1002/cnr2.70015)

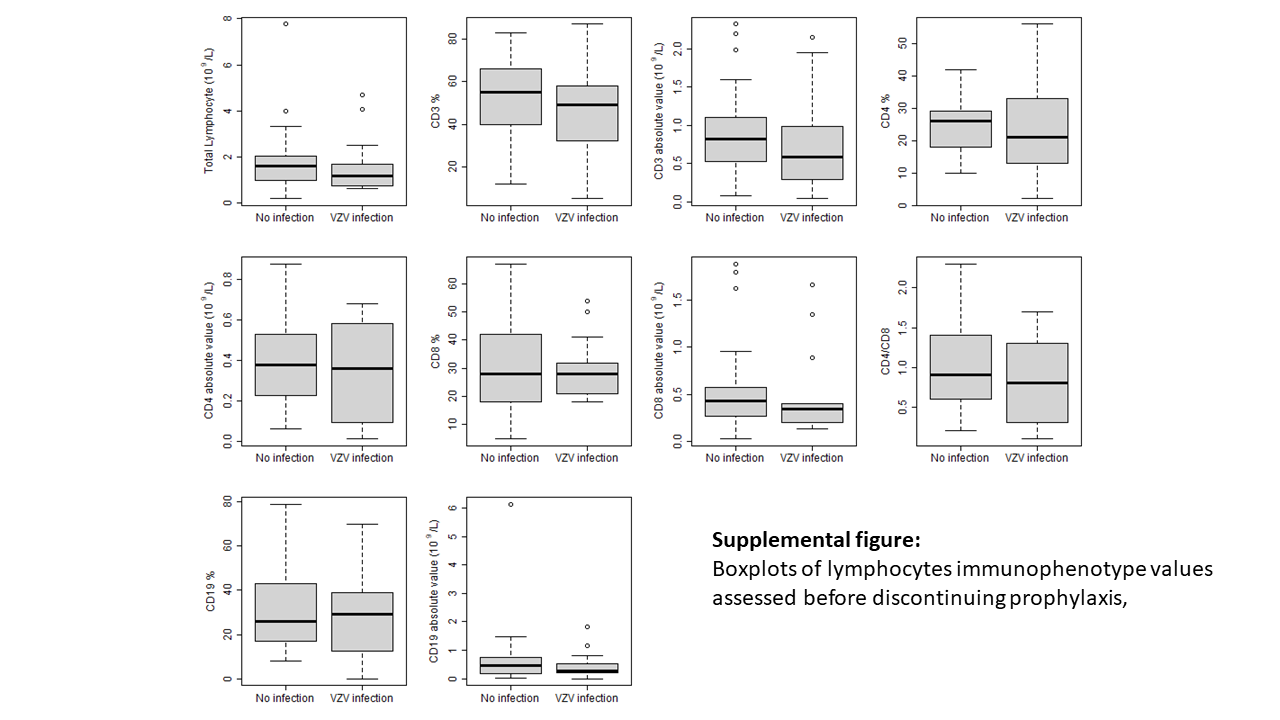

Supplement: Supplementary file 1 — Data S1. Boxplots of lymphocytes immunophenotype values assessed before discontinuing prophylaxis. [file CNR2-7-e70015-s001.png]
